# Human Factors and Data Logging Processes With the Use of Advanced Technology for Adults With Type 1 Diabetes: Systematic Integrative Review

**DOI:** 10.2196/humanfactors.9049

**Published:** 2018-03-15

**Authors:** Marion Waite, Clare Martin, Rachel Franklin, David Duce, Rachel Harrison

**Affiliations:** ^1^ Faculty of Health and Life Sciences Oxford Brookes University Oxford United Kingdom; ^2^ Faculty of Technology, Design & Engineering Oxford Brookes University Oxford United Kingdom; ^3^ Oxford Centre for Diabetes, Endocrinology & Metabolism University of Oxford Oxford United Kingdom

**Keywords:** adult, type 1 diabetes mellitus, T1D, technology, self-management, self-care, technology, telehealth, telemedicine, reminder system, continuous glucose monitoring, Sensor-augmented pump therapy, closed loop systems, adherence, compliance, barrier, usability

## Abstract

**Background:**

People with type 1 diabetes (T1D) undertake self-management to prevent short and long-term complications. Advanced technology potentially supports such activities but requires consideration of psychological and behavioral constructs and usability issues. Economic factors and health care provider capacity influence access and uptake of advanced technology. Previous reviews have focused upon clinical outcomes or were descriptive or have synthesized studies on adults with those on children and young people where human factors are different.

**Objective:**

This review described and examined the relationship between human factors and adherence with technology for data logging processes in adults with T1D.

**Methods:**

A systematic literature search was undertaken by using the Preferred Reporting Items for Systematic Reviews and Meta-Analyses (PRISMA) guidelines. Quality appraisal was undertaken and data were abstracted and categorized into the themes that underpinned the human factor constructs that were examined.

**Results:**

A total of 18 studies were included. A total of 6 constructs emerged from the data analysis: the relationship between adherence to data logging and measurable outcomes; satisfaction with the transition to advanced technology for self-management; use of advanced technology and time spent on diabetes-related activities; strategies to mediate the complexities of diabetes and the use of advanced technology; cognition in the wild; and meanings, views, and perspectives from the users of technology.

**Conclusions:**

Increased treatment satisfaction was found on transition from traditional to advanced technology use—insulin pump and continuous glucose monitoring (CGM); the most significant factor was when blood glucose levels were consistently <7.00 mmol/L (*P* ≤.01). Participants spent considerable time on their diabetes self-care. Logging of data was positively correlated with increasing age when using an app that provided meaningful feedback (regression coefficient=55.8 recordings/year; *P* ≤.01). There were benefits of CGM for older people in mediating complexities and fears of hypoglycemia with significant differences in well-being (*P* ≤.001).

Qualitative studies explored the contextual use and uptake of technology. The results suggested frustrations with CGM, continuous subcutaneous insulin infusion, calibration of devices, and alarms. Furthermore implications for “body image” and the way in which “significant others” impacted on the behavior and attitude of the individual toward technology use. There were wide variations in the normal use of and interaction with technology across a continuum of sociocultural contexts, which has implications for the way in which future technologies should be designed.

Quantitative studies were limited by small sample sizes, making it difficult to generalize findings to other contexts. This was further limited by a sample that was predominantly white, well-controlled, and engaged with self-care. The use of critical appraisal frameworks demonstrated where research into human factors and data logging processes of individuals could be improved. This included engaging people in the design of the technology, especially hard-to-reach or marginalized groups.

## Introduction

### Personal decision-making and human factors

Individuals with type 1 diabetes (T1D) are confronted with complex tasks through which to manage their blood glucose (BG) levels. T1D is an autoimmune disease where the beta cells in the pancreas no longer produce insulin, resulting in dangerously high BG levels or hyperglycemia. The person diagnosed with T1D is subsequently required to self-administer insulin. This involves regular self-monitoring of BG levels and calculation of appropriate insulin doses. There is a delicate balance between the reductions of the risks of long-term complications (often associated with hyperglycemia) and those of hypoglycemic events. This puts emphasis on adherence and patient behaviors. It has been suggested that large numbers of people with T1D are nonadherent [1]. Additionally, Patton [2] highlights multiple social, emotional, and cognitive barriers. The prevalence of new and emergent technologies to support self-management of T1D through personal data logging processes and support for decision making may have the potential to address these issues.

There may be a dilemma for health care providers due to the economic implications of adopting such technologies for individuals compared with potential public health benefits. This raises the issue of identification of adults with T1D who may benefit the most. There are associated questions around how to investigate and evaluate the benefits of such technology with respect to specific populations in such a way as to inform future design decisions. Thus, consideration of psychological and behavioral constructs alongside evaluation of the usability of devices, also known as human factors, is an integral component of any investigation that involves clinical consideration for emergent technology aimed at self-management of T1D.

The objective of this review was to describe the relationship between human factors and technology adherence for data logging processes in adults with T1D and to explore the factors that influence this association.

### Background

#### Advanced Technology for Self-Management of Type 1 Diabetes

The potential for technology to support individuals with T1D is increasing rapidly. The following overview covers general principles where the individual interacts with the technology to log his or her personal data in some capacity.

Continuous glucose monitoring (CGM) provides information regarding changes in glucose concentrations within interstitial fluid in real time. The corresponding device consists of a sensor, which is placed in the subcutaneous tissue, and a monitor, which may or may not be connected wireless. CGM data are used either to assist with retrospective decision making by a clinician or to support individual self-management. There is potential for an abundance of information about trends and directions in BG levels, including fluctuations over time for retrospective analysis [3]. One of the motivations for development of CGM is to recognize nocturnal hypoglycemia; another is to support people who may have lost their hypoglycemic awareness [4].

Real-time CGM has been available from 2005, and since then, advances in technology have improved the accuracy of CGM systems and provide potential advantages in terms of relaying the glucose history of an individual. Castle and Jacobs [5] suggest that there is valid evidence that both hyperglycemia and hypoglycemia are reduced with consistent CGM use. The optimal way to adjust insulin doses is complex, and there is little guidance for individuals about how to interpret the data. Internationally, there is low uptake of CGM but that may say more about availability and access than about the wishes of individuals.

Most individuals with T1D administer insulin via multiple daily injections (MDI), but some use an insulin pump that delivers bolus doses of insulin on demand of the user in addition to tiny amounts of insulin. These are administered every few minutes but may vary at different times of the day, thereby delivering what is known as continuous subcutaneous infusion of insulin (CSII). Advantages may include not physically injecting each delivered bolus dose and the availability of more physiologic basal insulin than available long-acting insulins can provide, and it is not necessary to inject each time a dose is administered. Theoretically, the way in which doses may be tailored is more specific to the insulin requirements of the individual [3]. There are 2 types of insulin pumps. One is tethered to a cannula that enters the subcutaneous tissues. This means that the pump must be worn by the user and may be visible. A patch pump on the other hand consists of a short tube attached to a cannula with an integrated micropump that is controlled wirelessly by the user [3], which can be hidden.

Sensor-augmented pump therapy (SAPT) is the concurrent application of real-time CGM with an insulin pump. However, this does not lead to automatic insulin adjustment. It is incumbent on the user to use adjunctive self-monitoring of BG and make dose adjustments to suit his or her own insulin requirements. Future developments include decision-support systems that will recommend insulin doses based on an array of factors, including historical data of the individual, and will also connect to health care providers.

Closed loop systems are sometimes known as artificial pancreas and manage insulin delivery in response to real-time CGM data, which is controlled by algorithms rather than preprogrammed rates [6]. According to Castle and Jacobs [5], this can also include delivery of glucagon to raise BG levels when necessary.

Apps run on mobile devices such as mobile phones and tablets and perform functions previously restricted to personal computers. Those designed specifically for people with T1D can generally be categorized into 5 areas:

1. Glucose tracking diaries
2. Carbohydrate estimators
3. Recipe planners
4. Medication adherence tools
5. Diabetes education platforms [7]

*Telehealth* refers to logging of health care data by the patient, which is tracked by health care professionals (HCPs) at a distance [8]. For example, the use of mobile devices by the patient enables any time, any place, anywhere logging and transmission of data.

#### Access, Uptake, and Current Limitations

Access and uptake of advanced technology, such as CGM and CSII, are controlled by health care economies and clinical policy guidelines. For example, in 2011, it was estimated that uptake may be between 20% and 30% in the United States and Israel compared with 1% in Denmark [9].

Acerini [10] claims that, even if CGM and CSII were readily available, those who could benefit the most from use would not access it and that diabetes technology uptake is lower in some ethnic groups. Furthermore, adoption is governed by socioeconomic status and cultural factors in addition to access to appropriate health care services. Crucially, health care practitioners’ willingness and capacity to support patient access are other critical factors [11].

To date, most research into use of advanced technology has focused on the clinical outcomes, which overall are equivocal [9,10,12]. Kerr and Partridge [6] critique the endpoints of previous clinical trials, which focus purely on glycated hemoglobin (HbA_1c_) levels without reference to other outcomes that may be equally meaningful to adults with T1D.

Transition and use of advanced technologies require training and physical and psychological adaptation by the users and their families. Human factors are, therefore, an essential component in reaching a better understanding of uptake and use of technology and in informing design decisions.

#### Human Factors and Type 1 Diabetes

There are differential aspects of the human factor that affect the use of technology in diabetes self-management [13]. These may be conceptualized as follows:

1. Behavioral
  - Barriers to adherence [2]
  - Demands of the technology, which may especially affect motivation to undertake regular self-management tasks [1,14]
  - Time spent on diabetes therapy tasks [11]
2. Psychological
  - Adjustment to diabetes [15]
  - Fear of hypoglycemia [11,14,16]
  - The emotional implications of increased responsibility for self-management including fear of disapproval by HCPs and worthiness to receive cutting-edge treatment [17]
  - Self-belief, impact on quality of life, reactions of others, unconscious motives based on earlier experiences [18]
  - Trust in the technology, letting go of prior routines [11,17]
  - Depression and eating disorders [18]
3. Social
  - Wearability of devices and body image [11]
  - Interpersonal relationships and working out how to handle interactions with others and when and how to disclose the condition [18]
  - Support from significant others to engage with technology [9]
  - Choice about whom to share data with [11]
  - Stigma surrounding the carrying out of tasks in social situations [4]
4. Cognitive
  - Educational needs, such as that of learning how to use the technology and utilize greater knowledge of personal glucose trends to make dosing decisions [9]
  - Additional learning associated with the use of technology [19]
  - Health literacy and associated embarrassment with low literacies [20]
  - Reduced cognitive abilities associated with age and adult level of educational attainment [21]

Current research in the field of advanced technology for diabetes has emerged from different disciplines, for example, health care practice, psychology, computer science, electronic engineering, and related industries. To reach a full understanding, it is crucial to bring this research together in a systematic way. Previous reviews have focused on clinical outcomes alone [5,22], have descriptively scoped the literature [13,23], or have synthesized studies on children and young people with studies on adults [24] where the needs for technology and associated human factors are likely to be different. Thus, there is a gap for a review that systematically appraises current research on the relationship between human factors and data logging processes with advanced technologies for adults with T1D.

### Aims of the Review

The aim of this systematic review was to describe the relationship between human factors and adherence with technology for data logging processes in adults with T1D and to explore the factors that influence this association.

An integrative literature review research design was chosen because it provides a more holistic conceptualization on a complex topic [25] such as human behavior and facilitates inclusion of diverse methodologies and theories, given the interdisciplinary approach toward research in the field.

A protocol was developed (Multimedia Appendix 1) to clarify the aims, sampling strategy, exclusion and inclusion criteria, methods, outcomes, language, and search strategy.

## Methods

### Literature Search

A systematic search of the literature was performed in accordance with the preferred reporting items for systematic reviews and meta-analyses (PRISMA) [26] in January to March of 2017 (Multimedia Appendix 2)

The following databases were searched: Computing Research Repository (2006 to January 2017); PsycINFO, EMBASE, and MEDLINE (2006 to January 2017); Web of Science (2006 to January 2017); Zetoc (2006 to January 2017); Excerpta Medica and Scopus (2006 to January 2017); and ProQuest (2006 to January 2017). Only research that was undertaken during the last 10 years was included as technology for the self-management of T1D has been developing rapidly during this time. Search terms included: Diabet* AND Techno* AND Behavi*; Self-manage* OR self-manage* OR manage* OR *self-care* OR self-care; technolog* OR *telehealth* OR *telemedicine* OR reminder system* OR text messag* OR *application* OR app*; adhere* OR *compliance* OR *barrier* OR problem* OR *obstacle*: MH Diabetes Mellitus, Type 1*.

Searches were limited to adults (over 18 years) and filtered to studies of adults published in English. Reference lists were also searched in addition to subject-specific websites and key journals (Multimedia Appendix 1). The search strategy was carried out in collaboration with a university health care librarian. Unpublished studies (dissertations and theses) were excluded, in addition to editorials, opinions, and discussion papers. Studies were reviewed for the following criteria: (1) primary research; (2) empirical data on adherence to data logging processes with the use of advanced technology for adults with T1D; (3) an investigation of the relationship with psychological, social, and human factors; and 4) the psychological outcome measures were explicit (quantitative studies) or alternatively included a clearly described picture of the phenomenon that included the user perspective (qualitative studies).

### Search Outcomes

The search strategy produced 1 article in the Computing Research Repository; 348 articles in PsycINFO, EMBASE, and MEDLINE; 40 articles in the Web of Science; 84 articles in Zetoc; 38 articles in Excerpta Medica and Scopus; and 36 articles in ProQuest. Once duplicates were removed, additional articles were excluded due to limitations associated with unclear abstracts or for not meeting the inclusion criteria (ie, children, type 2 diabetes, and gestational diabetes). In total, 72 citations were retained and each abstract was read for relevance.

Also, 3 citations were found from searching reference lists and key journals. One study, which included children and their carers, was retained because outcomes were compared with adults who also participated within the study [27]. To reduce bias and ensure that only the most relevant articles were selected, the second and third authors reviewed the titles and abstracts regarding the protocol criteria and a consensus was reached about the articles to be included in the review. In total, 22 articles met the inclusion criteria, and these included 14 quantitative studies, 5 qualitative studies, and 3 mixed-method studies (Multimedia Appendix 2).

### Quality Appraisal

Whittemore and Knafl’s approach [25] of using as many instruments as necessary to evaluate the quality of the data was taken because this is an integrative review, and the data are drawn from more than one disciplinary area that use a range of research traditions that align with quantitative, qualitative, or mixed-method research designs. The instruments for appraisal were selected from the University of South Australia International Centre for Allied Health Evidence [28] databases of critical appraisal tools. The following criteria were taken into consideration for types of study design: demographic information of the participants and statement of research question, appropriateness of the research question for the selected study design, and approach to recruitment reported (Table 1). The criteria for quantitative study designs included power analysis reported response rate, reliability and validity of study instruments and method of data analysis (Table 2). The following criteria were considered for qualitative studies: theoretical perspectives, audit trail, member checks, peer review of qualitative data, and method of data analysis (Table 3). The first author undertook the quality appraisal of each study, which was peer-reviewed independently by second and third authors.

Following the critical appraisal process, 4 studies were further excluded for poor methodological design.

### Data Abstraction and Analysis

The review data were categorized and synthesized into the themes that underpinned the human constructs that were examined and the outcomes that were reported. Mile and Huberman’s [46] approach to coding of data, which involves data reduction and comparison, was utilized.

**Table 1:** Quality appraisal.

Author	Type of study design	Aptness of study design for research aims	Demographic information of participants	Approach to recruitment reported
Groat et al [29]	Observational	No	Yes	Yes
Gonder-Frederick et al [30]	Observational	Yes	Yes	Yes
Skrosveth et al [31]	Observational	Yes	Yes	Yes
Tansey et al [27]	Randomized controlled trial	Yes	Yes	Yes
Kamble et al [32]	Randomized controlled trial	Yes	Yes	Yes
Martinez- Sarrigui et al [33]	Randomized controlled trial	Yes	Yes	Not reported
Gonzalez- Molero et al [34]	Longitudinal cohort study	Yes	Yes	Yes
Kirwan et al [35]	Randomized controlled trial	Yes	Yes	Yes
Polonsky et al [36]	Cross-sectional	Yes	Yes	Yes
Barnard et al [37]	Cross-sectional	Yes	Yes	Yes
Naranjo et al [38]	Cross-sectional	Yes	Yes	Yes
Borges and Kubiak [39]	Cross-sectional	Yes	Yes	Yes
Shepherd et al [40]	Qualitative	Yes	Yes	Yes
Ritholz et al [41]	Qualitative	Yes	Yes	Yes
O’Kane et al [42]	Ethnography	Yes	Yes	Yes
Storni [43]	Ethnography	Yes	Yes	Yes
Lawton et al [44]	Qualitative	Yes	Yes	Yes
Barnard et al [45]	Mixed methods	Yes	Yes	Yes

**Table 2:** Quality appraisal quantitative studies *.*

Author	Power calculation reported	Response rate (%)	Reliability and validity of study instrument established	Method of data analysis
Groat et al [29]	No	Not reported	No	Correlation analysis
Gonder-Frederick et al [30]	No	Not reported	Y^c^	Analysis of covariance
Skrosveth et al [31]	No	Not reported	No	Regression analysis
Tansey et al [27]	No	Not reported	Yes	Correlation analysis
Kamble et al [32]	No	Not reported	Yes	Correlation analysis
Martinez-Sarrigui et al [33]	No	Not reported	No	Regression analysis
Gonzalez-Molero et al [34]	No	Not reported	Yes	Correlation analysis
Kirwan et al [35]	Yes	Not reported	Yes	Regression analysis
Polonsky et al [36]	No	48.6	No	Regression analysis
Barnard et al [37]	No	96.7	No	Correlation analysis
Naranjo et al [38]	No	Not reported	Yes	Correlation analysis
Borges and Kubiak [39]	No	Not reported	Yes	Factor analysis

**Table 3:** Quality appraisal of qualitative research.

Author	Theoretical perspective	Audit trail	Member checks	Peer review of qualitative data	Method of data analysis
Shepherd et al [40]	Not reported	Yes	Not reported	Not reported	Thematic analysis
Ritholz et al [41]	Biophysical model of glycemic control in diabetes	Yes	Yes	Yes	Thematic analysis
O’Kane et al [42]	Sociocultural theory	Yes	Not reported	Not reported	Thematic analysis
Storni [43]	Ethnomethodology	Yes	Not reported	Not reported	Thematic analysis
Lawton et al [44]	Not reported	Yes	Yes	Yes	Thematic analysis

## Results

The 18 studies included in this review consist of 5 qualitative studies [40-44], 5 experimental studies [27,32-35], 3 observational studies [29-31], 4 cross-sectional studies [36-39], and 1 mixed-methods study [45]. Of the studies, 5 were smaller samples drawn from parent clinical trials [30,32,38,40,41].

The total number of participants who were included in the 18 studies was 3320 and the mean age was 42 years, although one study [36] specifically recruited people over the age of 65 years. Female participants represented 53% of the sample. The mean prebaseline HbA_1c_ was 7.9% (where reported).

Multimedia Appendix 3 summarizes the type of technology included in the review and the human factor constructs and outcomes that were examined.

After categorization and synthesis of themes, 6 overall constructs emerged:

1. The relationship between adherence to data logging and measurable outcomes
2. Satisfaction with the transition to advanced technology for self-management
3. Use of advanced technology and time spent on diabetes-related activities
4. Strategies to mediate the complexities of diabetes and the use of advanced technology
5. Cognition in the wild
6. Meanings, views, and perspectives from the users of technology

### The Relationship Between Adherence to Data Logging and Measurable Outcomes

There was inconclusive evidence about the relationship between adherence to data logging process and measurable outcomes. For example, Kirwan et al [35] examined a freely available iOS app—Glucose Buddy—combined with text messaging feedback from a diabetes educator aimed at the improvement of glycemic control. The intervention group showed a significant decrease in HbA_1c_ (mean −1.10; SD 0.74; (*P* ≤.001) over the 9-month period of the study; however, linear regression showed no significant relationship between the level of engagement with the app and these outcomes. This result may be interpreted with caution, given the small sample size (n=27). Furthermore, there was a potential socioeconomic bias in that participants were required to have iOS ownership.

Groat et al [29] analyzed individual participant internet protocol address data to characterize the relationship between adherence to insulin bolus dosing, logging of carbohydrate intake, and BG monitoring and glycemic control for a 1-month period. The only significant outcome was that an increase in daily insulin bolus doses had an impact on increasing the number of days that the BG was at target (*r*=.93). The reported results were based upon an extremely small sample (n=8) and described as regression analysis, which contradicts the researchers’ claims for undertaking a qualitative study.

### Satisfaction With Transition to the Use of Advanced Technology for Self-Management

Some findings suggest that adults with T1D may feel more satisfied with their treatment on transition to advanced technology. For example, Gonzalez et al [34] evaluated the overall effect of adding a telemedicine system for adults with T1D who were treated with an insulin pump and real-time CGM. This was a longitudinal study that measured the physical and psychological outcomes of the intervention. Mean plasma HbA_1c_ was significantly lower at 6 months compared with prebaseline (6.97 vs 7.5; *P*=.01); there was a significant reduction in glucose variability at 6 months compared with baseline (53.1 vs 68.7; *P*=.04) and prebaseline (53.1 vs 67.3; *P*=.04), and time spent interacting with the sensor correlated positively with time in normoglycemia (*r*=.72; *P*=.03) and negatively with occurrences of mild hypoglycemia (*r*=.64; *P*=.02). From a psychological perspective, there was an improvement in quality of life scores at 6 months in comparison with baseline (92.4 vs 86.9; *P*=.01), and participants with poorer glycemic control had significant improvements with prior dissatisfaction with treatment (34.3 vs 31.6; *P*=.01).

The authors acknowledged that the findings were based on a small sample size (n=15), and therefore, it is not possible to generalize the outcomes. The authors also questioned whether the point of being observed affected the outcome measures. However, the study did show that there may be benefits for well-controlled individuals using CGM in conjunction with telemetry in terms of HbA_1c_ and quality of life as reported in the previous paragraph.

There is some consistency regarding the perceived physical outcomes and satisfaction of the above study with the findings of Barnard et al [45], who measured the relationship between satisfaction when transitioning to the then-current insulin pumps (Animas Vibe CGM-enabled system IV) and personal glycemic control. The most significant contributing factor to treatment satisfaction was when BG levels were consistently <7.00 mmol/L (*P*=.009). The limits of this study are that the findings are based on self-report, and it is not clear why only 22 items of the 50 on the *Insulin* treatment satisfaction questionnaire were included on the survey instrument.

### Use of Advanced Technology and the Relationship With Time Spent on Device-Related Activities

Frequent users of existing diabetes technology may find an easier transition to more advanced options. For example, Tansey et al [27] examined the perceived barriers and benefits to CGM use and how this related to frequency of use. Engaged CGM users were more satisfied, with higher frequency users less bothered by the “hassles” of the device. Frequent users were classified as engaged with CGM for more than 6 days per week and infrequent users less than 4 days per week. Adults and parents of users had higher total and subscale scores on the CGM satisfaction survey (*P*=.0009). All respondents reported that visualization of glucose trends and the opportunity to detect hypoglycemia were the best aspects of use of CGM (text item responses in the questionnaire).

Martinez-Sarriegui et al [33] analyzed patient behavior when using the intervention of telemedicine system combined with CGM to identify how the CGM data captured participant interactions with the mobile system. In 2 phases of the experiment (with and without the telemedicine system), participants were provided with tools for visualization, management of monitoring data, and wireless downloading of data from an insulin pump via a personal smart assistant running on a personal digital device. The number of times interacting with the system was higher during the intervention phase (29.0 vs 18.8; *P*=.04), and the total time spent interacting with the system was also higher during the intervention phase (04:27:11 vs 01:47:07; *P*=.009).

Kamble et al [32] compared weekly estimates of time, changes in time, and patient time costs associated with diabetes-related care between SAPT and MDI. They used data on patient-reported time collected over a 52-week period. Participants were required to log the total time spent per week on diabetes management for a range of diabetes-related variables. The total time spent on the SAPT arm of the study was higher than time spent on MDI during and after pump initiation within the overall 52-week study. The reported weekly time estimates were as follows: SAPT 4.4 hours and MDI 3.4 hours (95% CI 0.4-1.7). However, all adults with T1D in the study reported that they spend considerable time on diabetes care.

Each of the above 3 studies suggests that engagement with technology is time consuming. Given that the inclusion criteria for the Tansey et al [27] study were prior high frequency of self-monitoring, it is not clear if the technology was a mediating factor for engagement. The Martinez-Sarriegui et al [33] study was limited by a small sample size (n=10) and did not include any details about how the study instrument was developed or how the participants valued the feedback from the telemedicine system. Furthermore, there was a possibility for margin of error with the Kamble et al [32] study as it was not clear how participants measured time costs.

### Strategies to Mediate the Complexities of Diabetes and the Use of Advanced Technology

Some researchers have attempted to understand the way in which the human complexities of diabetes have the potential to be mediated with the use of advanced technology.

#### Meaningful Feedback for the User

Skrosveth et al [31] explored which methods of diabetes data analysis could be realistically used to provide meaningful feedback for the user. A mobile diary app was developed for adults with T1D to log insulin doses and dietary intake with options for the user to comment upon these and a screen to visualize each of the following variables: BG level, insulin dosing, and dietary intake. Retrospectively, the sample was divided into 2 groups: “adopters” (n=18), who reliably logged data for at least 80 days, and *nonadopters* (n=12), who did not. Logging of data was positively correlated with increasing age (regression coefficient=55.8 recordings per year; *P* ≤.007), but the usage did not significantly correlate with prestudy HbA_1c_ (*P*=.33) or gender (*P*=.09). The researchers also found that several methods of pattern recognition were unable to predict future BG values. The study was limited by lack of demographic information about the participants and how they were recruited. More information about *nonadopters* such as confounding variables would have increased the reliability and validity of the results.

#### Engaging Older Adults With Continuous Glucose Monitoring

Polonsky et al [36] surveyed 2 groups of participants aged 65 years and older with T1D to determine differential characteristics between users of real-time CGM and nonusers (*hopefuls*). CGM *hopefuls* reported a higher incidence of 1 moderate hypoglycemic episode in the preceding 6 months (90% vs 78%; *P*=.04), 1 hypoglycemic-related emergency room visit during the preceding 6 months (18.7% vs 6.7%; *P*=.002), and 1 hypoglycemic event requiring assistance by another during the preceding 6 months (80% vs 57.6%; *P* ≤.001). CGM *hopefuls* also reported significant differences in well-being (*P*=.009), hypoglycemic distress (*P*=.04), and feeling of powerlessness (*P*=.04). The study suggested potential benefits for older people with the use of advanced technology, which is important given that hypoglycemic unawareness increase with age. A drawback of the study was that the 2 groups were of unequal sizes: the user group=11 and the *hopeful* group=75.

#### Information Overload and Ease of Use

Borges and Kubiak [39] explored the relationship between information overload, ease of use, and personal attitude in the use of CGM by identification of motivations to use CGM and comparison of characteristics between groups with differing levels of CGM experience. The findings were that, irrespective of the level of experience, the advantages of CGM were perceived as high and the disadvantages perceived as low. There was a significant difference with respect to perceived information overload; adults with T1D without experience rated this higher than adults with T1D with more experience (90% CI 1.443-0.785; *P* ≤.001). This is important because information overload had a negative influence on the ease of use (*P* ≤.001). The study reports statistically significant outcomes; however, the participants were recruited through Web-based forums and social media and described as having high levels education, which was a potential socioeconomic limiting factor.

#### The Potential of Continuous Glucose Monitoring to Explore Stressors

Gonder-Frederick et al [30] investigated the relationship between routine daily stressors, BG levels, and diabetes management strategies in a naturalistic setting using a CGM data to generate BG profiles (adults with T1D were also participating in multicenter cross-over randomized controlled trial closed- loop control CLC study). There was no relationship found between stress ratings and average daily glucose. However, stress ratings were positively related to low BG levels (*P*=.025). Overall, the results suggested individual differences between stress and glycemic control for people with T1D and the potential of CGM to explore this more in depth. This needs to be countered with the acknowledged small sample of participants (n=33) and a short-term study with highly selected participants.

#### The Relationship Between Diabetes Distress and Technology

Naranjo et al [38] undertook a comparative analysis of the level of diabetes distress that is associated with diabetes devices and technology between users of traditional technology (BG meters and MDI) and advanced technology (pump therapy and CGM). The results showed significant differences between attitudes to technology with CGM users being more positive than nonusers (24.87 vs 23.87; *P* ≤.001). Pump users were more positive than MDI users (24.8 vs 22.98; *P* ≤.001). There were no significant differences in distress across all types of technology use by participants. However, there was no account for confounding variables other than age.

Ritholz et al [41] qualitatively compared psychosocial differences between 3 groups of participants who were participants from the Juvenile Diabetes Research Trial: *responders* (n=7), drawn from a primary cohort who had shown improvement in glycemic control; *responders* (n=6), drawn from a secondary cohort who had demonstrated a reduction in HbA_1c_ in within target range, and *nonresponders* (n=7), who had a less than 0.5% reduction in HbA_1c_. The following themes emerged from the findings: *coping with frustrations*, *use of CGM information*, *significant other information,* and *body image*. Frustrations were experienced with CGM, CSII, calibrations, and alarms. *Responders* reported a *self-controlling coping style* whereas *nonresponders* were more likely to make an emotional response. All participants were engaged with minute-to-minute information, but *responders* were more likely to use retrospective information to spot trends and act upon them. Many *responders* reported *significant other* involvement, especially males who suggested that this allayed other important fears about the risks of hypoglycemia. Body image of use of the device was associated with “nonresponders,” who felt uncomfortable about using the device in public places and intimate situations. The researchers raised the role of “significant others” in CGM research and suggested that this is an underexplored area. The research also highlights the clinical implications of preparation of adults with T1D to deal with frustrations and cognitive overload.

The limitations of the research are that it was carried out on a population that was described as well educated and homogenous.

### Cognition in the Wild

Some researchers have adopted an ethnographic approach to explore how technology is used in the context of the everyday lives of adults with T1D.

O’Kane et al [42] took a sociocultural perspective and reported on 3 qualitative studies that examined how devices for adults with T1D are adopted, carried, and used in a variety of everyday contexts. This is based on the premise that adults with T1D are encouraged to self-regulate by HCPs, but the nature of everyday life is contingent upon the dynamics of the unfolding situation. The following themes emerged from the data analysis: *misuse*, *inappropriate use*, and *unintended use* of the technology. The authors’ main point is that any individual can report a wide variation in normal use of their technology across a continuum of public use, work-life use, and in the company of friends and family. This was based on the perceived emotions and attitudes of the other party within a given context. Uncertainty in discrete situations can lead to hiding a device, whereas showing off the device in other situations can lead to normalization and control of a situation. This corresponds with the findings of Ritholz et al [41], which were reported in the previous section regarding the place of *significant others* in uptake and use of technology.

### Meanings, Views, and Perspectives From the Users of Advanced Technology

Research that examines the meaningfulness and perspectives of the user has an important role to play in the future and ongoing development of advanced technology. Shepherd et al [40] explored both desires and concerns regarding the use of CGM for self-management. The findings suggested that adults with T1D who already used insulin pumps and CGM had a diverse range of attitudes and concerns along a continuum regarding personalized glucose advisory systems. Participants would have liked advice from the system on suggestions for correction boluses, basal rates, insulin-carbohydrate ratios, and alerts to the risks of hypoglycemia. However, it would be necessary for the individual to understand how the advice was generated, trusting that all personal variables would be considered to develop the confidence to relinquish control to an automated system. A shortcoming of the study is that it was not entirely clear how the themes were arrived at.

Lawton et al [44] (2014) found evidence of similar themes during a longitudinal study of the use of insulin bolus calculators following the intervention of a dose adjustment for normal eating course. Adults with T1D were motivated by the device because it saved time and effort in calculations; however, those who were confident in their mathematical ability undertook their own individual calculations and were paradoxically less likely to use the device over time. Reliance on the calculator alone had a detrimental impact on glycemic control. Some participants left the ratios unchanged until their next clinician/study review, and for some, this was attributed to not knowing how to change the settings. Underconfidence in carrying out personal calculations or not knowing how to change settings led to loss of trust in the technology.

Storni [43] contends that diabetes is more than a disease and should be regarded as a complex lifestyle. People with T1D develop lay expertise that is unique to their situation. This creates implications for technology design, and it is crucial to involve the user in the process. This perspective is based upon findings that emerged from an ethnographic study on diabetes support groups and by following individuals with T1D within the context of their everyday lives [43]. The purpose was to examine what participants really did in dealing with their condition as opposed to what they were told to do by clinicians. These findings influenced the design of a tagging system for events from everyday life to link them to carbohydrate intake and BG readings to create meaning between the events and a log for the individual on a mobile device. A shortcoming of the study is that the report provided a lack of demographic information about participants, which is important in qualitative research to determine transferability to other contexts. Nevertheless, there is an emergent field of research that addresses the diverse needs of people with T1D in the design of technologies.

## Discussion

### Principal Findings

Advanced technology for the management of T1D needs to have clear benefits that are meaningful to adults with T1D. The aim of the review was to describe the relationship between human factors and adherence with technology for data logging processes in adults with T1D and to explore the factors that influence this association.

There was inconclusive evidence about the relationship between adherence to data logging and measurable outcomes in relation to the review question. However, clinical values may have less importance than perceived outcomes for individuals. The review did suggest increased satisfaction with treatment on transition to advanced technology; however, this was biased toward frequent users of existing technologies and with an acceptance of the time required to spend on diabetes care.

The review also showed some benefits of advanced technology for older people by mediating complexities and fear of hypoglycemia. There appears to be a wide variation in the normal use of technology for adults with T1D across a continuum of sociocultural contexts. There is also a variability regarding user involvement in the design of future technologies and the role of “significant others” and this requires further research. People need to be able to trust technology as the capacity for intelligent decision-making advances.

In the literature that was reviewed, participants appeared to be a highly selective group biased toward white populations. Another limitation was the relatively small sample sizes of some of the quantitative studies included within the review, only 1 study [35] reporting on a power calculation, thus making it hard to generalize the findings.

A significant issue was that where demographic characteristics were reported (Table 1), 95% of the participants were described as white. The data suggest that those from higher socioeconomic groups are more likely to have access to and engage with technology in their self-management behaviors [38]. Of the studies, 2 [30,45] purposefully selected participants with prior adherent behaviors; however, 1 study recruited participants who were less engaged with technology and adherence [32].

The predominance of white participants, combined with the fact that 6 of the reviewed studies were samples drawn from parent clinical trials, suggests that the data are based on a highly selective group. This may not be representative of the general adult population with T1D. The mean baseline HbA_1c_ of 7.9 implies that participants had relatively good control before entering one of the respective studies, which may suggest a largely adherent sample.

Although qualitative research is not considered to be necessarily generalizable by some audiences [47], it is incumbent on the researcher to provide full demographic descriptions so that the generic reader from an interdisciplinary audience can decide about the transferability of findings to his or her own practice, research, or development context. Furthermore, trustworthiness of the findings can be clarified based on participants’ checking of data and peer review of data analysis. This was a shortcoming of some of the literature that was reviewed.

### Implications for Health Care Practice

Engaged participants spend considerable time on diabetes care, so it is important that they receive support to make informed choices. On the basis of this review, it was found that these are the people most likely to benefit from the affordances of advanced technology; however, this creates a tension between these populations and hard-to-reach groups who may be at increased risk of diabetes complications. Furthermore, Lawton et al [44] suggest that in general HCPs lack knowledge about the scope and purpose of advanced technology for diabetes. This is important, given the potential information overload and the frustrations that adults with T1D are presented with when using technology demonstrated within this review and other literature [9,19,41].

What is meaningful for the adult with T1D might not be important for the clinician and may therefore require mediation. Storni [43] found that patient-generated tags for mobile devices developed by participants were not of interest to clinicians who were more focused on numerical values.

James et al [47] have explored the perceptions and experiences of diabetes educators when supporting the use of advanced technology and suggest that there are challenges for all parties. This includes device costs, access to Wi-Fi, and appropriate mobile devices. CSII puts demands on diabetes services, and there are also challenges associated with keeping up to date with technology, such as the skills to analyze data from patient mobile devices. This research study suggested that there is a need for mentorship of HCPs and a review of service configurations as technology advances.

### Implications for Future Design of Technologies

Engaging people in the design of technology for T1D is essential for meeting the requirements of the user. Within this review, O’Kane et al [42] suggested that the design of devices needs to be both discrete and more public for context-dependent behavior. Lawton et al [44] suggested that voice recognition for entering data would make data logging practices easier for some people. Engaged participants appeared to be able to deal with the hassle and time required for diabetes-related tasks. However, a challenge for designers is to build in time-efficient capabilities.

### Implications for Future Research

There is a requirement for studies within the context of day-to-day data logging that are representative of the general adult population with T1D. There is more scope for research that explores how technology could be used to engage hard-to-reach groups. Many of the studies in this review were short-term; however, the study undertaken by Lawton et al [44] on the use of insulin bolus calculators was in-depth and over time (1 year), thus providing a rich and diverse view of adherence and nonadherence along a trajectory, which provided important nuances about human factors. There is also a need to study the role of *significant others* within data logging processes [41]. There appeared to be a dearth of mixed-methods studies, which if conducted through a rigorous methodological process have the potential to capture the complexity of human factors by maximizing the advantages of more than one research design. There is also a need for future studies that explore the sociocultural and demographic factors associated with technology uptake.

### Limitations

A limitation of this review is that the data were drawn from databases, which excludes emergent unpublished research in a fast-moving field. However, this was mitigated by extracting the data from sources retrieved from 9 key databases covering the fields of health, medicine, and computer science, and the search was performed in collaboration with a university health care librarian.

### Comparison With Prior Work

The application of critical appraisal frameworks used in this review made it possible to evaluate the reliability, validity, and trustworthiness of each of the studies under consideration. This review presents a contribution to the field in comparison with descriptive mapping reviews and highlights areas where research design could be improved. By abstracting data from each of the studies, it was possible to compare the findings and focus on the human factor constructs of adult populations with T1D, including older people.

### Conclusions

The purpose of this systematic review was to explore the relationship between human factors and the adherence to technology for data logging in adults with T1D. The research design was an integrative review, given the interdisciplinary nature of research in the field and the diverse methodological approaches taken to inquiries.

The aim of the review was to analyze the relationship between human factors and adherence to technology for data logging in adults with T1D. Overall, the sample was drawn from homogeneous populations that may not be the complete representation of adults with T1D. Inconclusive evidence was found about the relationship between adherence to data logging with advanced technology and measurable outcomes. There was some suggestion that adults with T1D may feel more satisfied with their treatments on transition to advanced technology. Qualitative research suggested that the way in which technology is used by any individual varies along a continuum and is contingent upon the sociocultural context in which technology is used. As technology continues to advance, there is a need for more research into how trusting the individual is of personal treatment advice, which is generated through advanced technology.

## Appendix

Multimedia Appendix 1 Systematic review protocol.

Multimedia Appendix 2 PRISMA flow diagram.

Multimedia Appendix 3 Data abstraction, technology, and human factors.

## Abbreviations

BG: blood glucose
CGM: continuous glucose monitoring
CSII: continuous subcutaneous insulin infusion
CLC: closed loop control
HbA_1c_: glycated hemoglobin
HCP: health care professional
MDI: multiple daily injections
PRISMA: Preferred Reporting Items for Systematic Reviews and Meta-Analyses
SAPT: sensor-augmented pump therapy
T1D: type 1 diabetes
