# Supplementary material for: Human Factors and Data Logging Processes With the Use of Advanced Technology for Adults With Type 1 Diabetes: Systematic Integrative Review
Source: JMIR Hum Factors. 2018 Mar 15;5(1):e11. doi: 10.2196/humanfactors.9049 (PMC5871738; doi:10.2196/humanfactors.9049)
Supplement: Multimedia Appendix 1 [file humanfactors_v5i1e11_app1.pdf]

Multimedia Appendix 1 Systematic Review Protocol

Research question

In adults with Type 1 diabetes, what is the relationship between psychosocial factors and adherence with technologies for data logging processes?
The research question is framed within participant experiences of the use of technology and the impact of advanced technology for diabetes self-management.

Exclusion: Children and adolescents with T1DM or T2DM; Adults with T2DM or gestational diabetes; empirical studies more than 10 years old, non-empirical data.
Inclusion:
Sample: Adults with T1DM.
Methods International research undertaken during the last 10 years, systematic reviews, quantitative studies (experimental, quasi-experimental, non-experimental), qualitative studies and mixed methods.
Outcomes: Use of advancing technologies that may impact on self-management behaviour.
Language: Studies that are published in English.

Search strategy (databases, websites, journals, personal contacts)

Databases: Cochrane Collaboration, Medline, Embase, AMED, Cinahl, PsychoINFO, International Bibliography of the Social Sciences, Computing Research Repository, ZETOC, Web of Science, Sociological Abstracts, Scopus, Global Health, Science & Technology Proceedings, Technology Research Database, ACM Digital Library.

Websites: Johanna Briggs Institute, Diabetes UK.

Journals: Journal of Diabetes, Science and Technology, Diabetes Technology & Therapeutics, Diabetic Medicine, Primary Care Diabetes Europe.

Personal Contacts: Dr Jose Manuel Fernandez-Real (IDIBGI, Spain), health care subject librarian

Multimedia Appendix 1 Systematic Review Protocol

Table 1: Search Strings, (synonyms, combinations, wildcards and brackets)

	Search ID#	Search Terms
☐	S19	S1 AND S2 AND S8 AND S16
☐	S18	S1 AND S2 AND S8 AND S16
☐	S17	S1 AND S2 AND S8 AND S16
☐	S16	Self-manage* OR self manage* OR manage* OR self-care OR self care
☐	S15	Self-manage* OR self manage* OR manage* OR self-care OR self care
☐	S14	S1 AND S2 AND S3 AND S8
☐	S13	S1 AND S2 AND S3 AND S8
☐	S12	S1 AND S2 AND S3 AND S8
☐	S11	S1 AND S2 AND S3 AND S8
☐	S10	S1 AND S2 AND S3 AND S8
☐	S9	S1 AND S2 AND S3 AND S8
☐	S8	technolog* OR telehealth Or telemedicine OR reminder system* OR text messag* OR application OR app*
☐	S7	S1 AND S2 AND S3 AND S5
☐	S6	educat* technolog*
☐	S5	technolog*
☐	S4	teachnolog*
☐	S3	Self-manage* OR self manage* OR manage*
☐	S2	Adhere* OR compliance OR non-compliance OR non compliance OR barrier OR problem* OR obstacle
☐	S1	(MH "Diabetes Mellitus, Type 1")
