# Supplementary figures and images for: Human Factors and Data Logging Processes With the Use of Advanced Technology for Adults With Type 1 Diabetes: Systematic Integrative Review
Source: JMIR Hum Factors. 2018 Mar 15;5(1):e11. doi: 10.2196/humanfactors.9049 (PMC5871738; doi:10.2196/humanfactors.9049)

## Multimedia appendix 2, Prisma flow diagram

Prisma Flow Diagram[26]

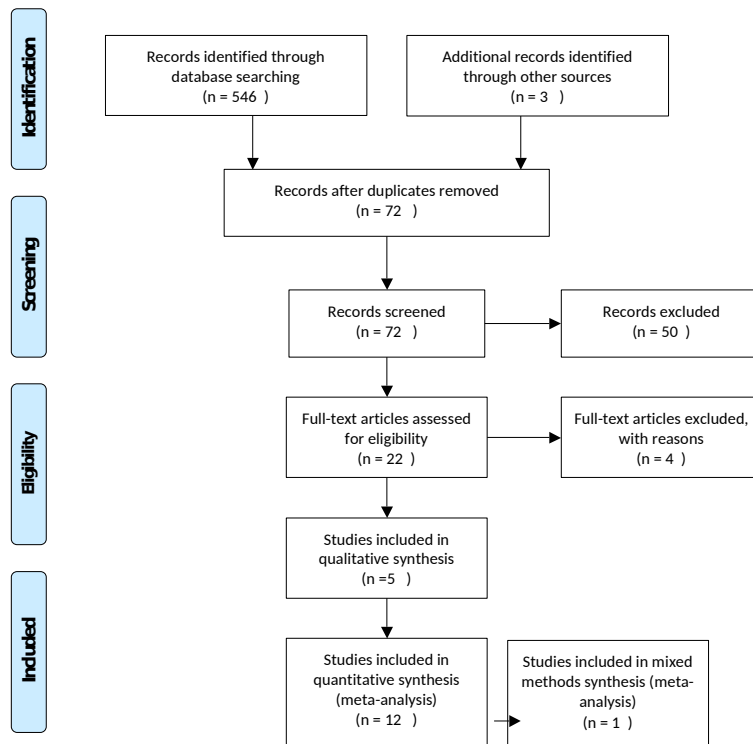

Supplement: Multimedia Appendix 2 [file humanfactors_v5i1e11_app2.pdf]
