# Supplementary material for: Human Factors and Data Logging Processes With the Use of Advanced Technology for Adults With Type 1 Diabetes: Systematic Integrative Review
Source: JMIR Hum Factors. 2018 Mar 15;5(1):e11. doi: 10.2196/humanfactors.9049 (PMC5871738; doi:10.2196/humanfactors.9049)
Supplement: Multimedia Appendix 3 [file humanfactors_v5i1e11_app3.pdf]

Multimedia appendix 3, data abstraction, technology and human factors

Table 1 Data abstraction, technology and human factors

	Groat et al [28]	Gonder-Frederick et al. [29]	Skrosveth et al. [30]	Tansey et al.[31]	Kamble et al. [32]	Matinez-Sarriegui et al. [33]	Gonzalez-Molero et al. [34]	Kirwan et al. [35]	Polonsky et al. [36]
**Setting**		USA, seven centers, Italy, one center.	Norway	USA, three centers.	USA	Spain, three centers.	Spain, five centers.	Australia.	USA, four centers.
**Sample size**	8	33	30	224	483	10	15	27	296
**Technology**	IP data analysis (Medtronic) of relationship between insulin bolus dosing, CHO intake and BG monitoring CGM & Capillary Glucose Monitoring.	CGM data, Closed Loop Artificial Pancreas.	Mobile phone App (Few Touch Application) and feedback.	CGM, cross-over CGM and standard blood capillary monitoring.	SAP vs. MDI.	Telemedicine (DIABTel) & CGM via the use of a PDA vs. SMBG (Roche Acutrend).	Telemedicine added to RT-CGM & insulin pump. Physician response within 48 hours.	Mobile phone input and feedback app iOS "Glucose Buddy", weekly text messaging support for MDI or pump.	CGM uptake and use over 65 years of age.
**Human factor construct**	Adherence to: 1) CHO counting x 3/day 2)Delivery of insulin bolus X 3/day 3) Checking BG x 4/day. Comparison of adherent days with non-adherent days in relation to when BG was on target	Daily stressors to predict BG, CHO and insulin intake, diabetes self-management strategies.	Use of the App in relation to age and pre-study Hba1c.	Frequency of use of CGM monitoring associated with perceived benefits and barriers.	Patient time costs on diabetes related care.	Number of interactions with the system compared between interaction and control, CGM compared to no CGM.	Number of self-controls, time with sensor in relation to glycemic control and QOL.	Engagement with the app and relationship with diabetes outcomes.	Comparison of CGM use with anticipated CGM use correlated with demographic factors, hypo experiences, fear of hypo and diabetes distress.
**Data collection method**	Downloaded IP data, CHO & BG levels	CGM and pump data correlated with daily diaries of	Downloaded data from mobile phones and feedback	Continuous Glucose Monitoring Satisfaction Scale,	52 week diaries of recorded time spent per week on diabetes	Tracking of user interactions, online questionnaire, utility of	Diabetes quality of life (Spanish version), DQOLPR, social/voc	Diabetes Empowerment Questionnaire (DES-SF), Summary	Survey to examine quality of life and health benefits of RT-

		recorded stress levels.	on user interface.	CGM-SAT.	management.	the CGM device for decision-making	ational concerns, (DQOLINSAT,.). Treatment dissatisfaction, (DQOLIMP). Impact of the treatment, FH fear of hypoglycaemia, STAIE, Anxiety sate, STAIR, anxiety traits, BDI, depression, BMI, B/P, review of meter data, FBG, Hba1c	f Diabetes Self-care Activities (SDSCA), Diabetes Quality of Life (DQOL)	CGM in seniors.
**Key findings**	Adherence to 3 behaviors was similar between CGM and capillary. Increasing the daily insulin bolus had greatest impact on number of days that BG was on target, *r* 0.93	Stress ratings positively correlated WITH low BG , *r* = .153, n =218, *P* = 0.35, *r*2 =2.33%	Positive correlation with age, *R* =56.8 recordings per year(*P* =0.0008), usage not significantly correlated with pre-study HBA1c (*P* = 0.33), or gender (*P* =0.09)	Adults and Parents of Youths had higher total subscale scores (*P*= 0.0009)	Following pump initiation, SAP =4.4 hours, MDI = 3.4 hours (95% CI 0.4-1.7).	Insulin pump therapy visualizations were used most significantly during the intervention phase 29.0 vs. 18.8 (*P* =.04).	Well controlled individuals with T1DM who adhere to CGM & pump therapy benefit from telemetry in terms of hba1c and QOL, Hba1c significantly lower at 6/12 (6.97) compared with pre-baseline (*P*+0.010) , improvement in QOL at 6/12 compared to baseline 92.4 vs. 86.9 (*P* = .011)	Intervention group had a significant decrease in mean HbA1c (mean = -1.10, SD 0.74 *P* = 0.0009 over 9/12 study, significant change in diabetes self-care measure of specific diet over time 6.90 *P* = 0.04.	"Hopefuls" more likely to report moderate hypo in pre-ceding 6/12, 90.7% vs. 78.1%, (*P* =. 04)

Multimedia appendix 3, data abstraction, technology and human factors

IP = , CHO= carbohydrate, BG= blood glucose, CGM= continuous glucose monitoring, SAP = sensor augmented pump therapy, MDI= multi-dose injections, PDA = personal digital assistant, SMBG = self-monitoring of blood glucose levels

	Barnard et al. (2015b) [37]	Naranjo et al. (2016) [38]	Borges and Kubiak (2016) [39]	Shepherd et al. (2012) [40]	Ritholz et al. (2010) [41]	O'kane et al. (2015). [42]	Storni (2015) [43]	Lawton et al (2014) [44]	Barnard et al. (2015a)
**Setting**	France, Germany, Netherlands and UK, 141 centers.	USA, two centers	Germany, one center	USA, seven centers, Italy one center	USA, two centers	London, Los Angelos, Toronto	Ireland, one center	Scotland, one center	USA, one center, UK five centers
**Sample size**	361	1503	111	56	20	41	14	42	24
**Technology**	Insulin pump therapy (Animas Vibe CGM-enabled system IV)	Insulin pumps, CGM & SAP	CGM	PGA system and CGM	CGM	All diabetes technologies available in the everyday context (at least a BG meter)	Tag it yourself mobile application	Automated insulin bolus calculator	Closed loop and open loop technology.
**Human factor construct**	Satisfaction with the device in relation to glucose control and insulin delivery. BG levels correlated with educational status	Physical, social, cognitive and affective distress esponder with diabetes devices and up take of technology.	Information overload, ease of usefulness and relationship with attitude	Desire to use CGM for self-management, concerns about advice generated, esponder to use of CGM	Comparison of three groups from the JDRF, for attitudes towards CGM use: esponder (improved glycemic control); esponder (decreased hypos and at target HbA1c);and non-responders (<0.5% decrease in HbA1c)	Use of technologies in the context of everyday lives	Self-care practices with technologies available in the everyday context.	Use of an insulin bolus calculator over time following DAFNE intervention Motvations for use, initial experiences, dependency and deskilling, reasons for stopping use.	Satisfaction with the technology, perceived health outcomes and ease of use of the device at end of cross-over intervention.
**Data collection method**	Insulin Treatment Satisfaction Questionnaire (ITSQ) 22/50 items recorded	Diabetes Distress Scale (DDS-TI, Diabetes Technology Attitudes (DSAT)	German translation of technology acceptance model (TAM)	Qualitative focus group interviews	Qualitative interviews.	Contextual interview, diabetes diary to record the moments that technolo	Participant observations during diabetes support groups, interviews, participat	Qualitative interviews	Diabetes Technology Questionnaire (DTQ), semi-structured qualitative

	.					gy was used, and participant observation during a tech meet-up	ory design workshop.		interviews
**Main outcomes**	Most significant contributing factor was treatment satisfaction when the BG was <7.00mmol/L (*P*= .0009)	Participant uptake of CGM and Pump therapy significantly older than non-users 38.29 vs. 33.48 *P* =.009	Significant relationships between: information overload and ease of use (95% CI 1.443-0.785 , *P*= .0009), Ease of use to usefulness (CI 0.297-1.077 *P*= .0009), Attitude to Intention (CI 0.058-1.528, *P* =.004)	Those most highly motivated in their self-management strategies less likely to hand over control to automated systems.	Coping with frustrations, use of personal CGM information, significant others, body image	Wide variation in normal use of technology, public use, work-life use, use around significant others, uncertainty in certain situations, hiding and showing off devices.	Diabetes represents complex lifestyle, patients develop lay expertise, importance of user involvement in the technology design process.	Motivation to use Bolus calculator, intial experiences, dependency and deskilling, reasons for ceasing to use	Satisfaction with the technology was the same for both groups (*P* = .15), qualitative themes technology lived up to expectations, participants felt 'normal' using the technology and sense of loss at end of trial, improved reported sleep & BG levels, usability issues obstrusive alarms, size of devices and connectivity.

PGA = Personal glucose advice, DAFNE (Dose adjustment for normal eating).
